# Tumor cell-derived PDGF-B potentiates mouse mesenchymal stem cells-pericytes transition and recruitment through an interaction with NRP-1

**DOI:** 10.1186/1476-4598-9-209

**Published:** 2010-08-05

**Authors:** Kakali Dhar, Gopal Dhar, Monami Majumder, Inamul Haque, Smita Mehta, Peter J Van Veldhuizen, Sushanta K Banerjee, Snigdha Banerjee

**Affiliations:** 1 Cancer Research Unit, VA Medical Center, 4801 Linwood Blvd, Kansas City, Missouri 6 4128, USA; 2 Division of Hematology and Oncology, 2330 Shawnee Mission Pkwy, Westwood, Kansas 66205, USA; 3 Department of Anatomy and Cell Biology, University of Kansas Medical Center, 3901 Rainbow Blvd, Kansas City, Kansas 66160, USA

## Abstract

**Background:**

New blood vessel formation, or angiogenic switch, is an essential event in the development of solid tumors and their metastatic growth. Tumor blood vessel formation and remodeling is a complex and multi-step processes. The differentiation and recruitment of mural cells including vascular smooth muscle cells and pericytes are essential steps in tumor angiogenesis. However, the role of tumor cells in differentiation and recruitment of mural cells has not yet been fully elucidated. This study focuses on the role of human tumor cells in governing the differentiation of mouse mesenchymal stem cells (MSCs) to pericytes and their recruitment in the tumor angiogenesis process.

**Results:**

We show that C3H/10T1/2 mouse embryonic mesenchymal stem cells, under the influence of different tumor cell-derived conditioned media, differentiate into mature pericytes. These differentiated pericytes, in turn, are recruited to bind with capillary-like networks formed by endothelial cells on the matrigel under *in vitro *conditions and recruited to bind with blood vessels on gel-foam under *in vivo *conditions. The degree of recruitment of pericytes into *in vitro *neo-angiogenesis is tumor cell phenotype specific. Interestingly, invasive cells recruit less pericytes as compared to non-invasive cells. We identified tumor cell-secreted platelet-derived growth factor-B (PDGF-B) as a crucial factor controlling the differentiation and recruitment processes through an interaction with neuropilin-1 (NRP-1) in mesenchymal stem cells.

**Conclusion:**

These new insights into the roles of tumor cell-secreted PDGF-B-NRP-1 signaling in MSCs-fate determination may help to develop new antiangiogenic strategies to prevent the tumor growth and metastasis and result in more effective cancer therapies.

## Background

Tumor cells assign neighboring blood vessels to support their own blood supply for oxygen and nutrients and finally for intravasation (to enter into the blood vessels) and extravasation (metastatic spread) through promoting pathologic neovascularization/angiogenesis [1-3]. This event is potentiated by tumor cells through the production of diffusible angiogenic factors [4-6]. New blood vessel formation/angiogenesis and remodeling of the vessel is a complex event and is dependent on proliferation, differentiation, mobilization and attachment of endothelial cells (ECs) and mural cells (MCs) with different phenotypic variants such vascular smooth muscle cells (VSMCs) and pericytes (PCs) in an autocrine-paracrine manner [7-11]. The literature on the molecular interactions of tumor cells with ECs for the angiogenic switch is appreciable, but less is known about mural cells.

VSMCs/PCs, which are located in different vascular systems according to their needs [11], play critical roles in both normal and pathologic vascular development, integrity and its maintenance [11-14]. Although VSMCs and PCs are morphologically similar, and express common molecular markers, they may function differently [11]. The vascular SMCs provide structural support to the large vessels and are critical regulators of blood flow, while PCs appear to be involved in the early events of capillary sprouting. The PCs are regularly found lying at and in front of the advancing tips of endothelial sprouts and may serve as a guiding structure of endothelial outgrowth [11,12] and termination of the event [13]. PCs are irregular in shape in tumors and loosely associated with ECs on tumor vessels [15,16], During new blood vessel formation and assembly, recruitment of PCs through the differentiation of precursor cells (mesenchymal), migration and attachment to the newly formed capillaries are vital events of this multistep process [17,18]. However, the role(s) of tumor cells in differentiation, recruitments and attachment of these cells are still under described. Therefore, we are interested to explore whether the tumor cells have the ability to differentiate, recruit and interact with PCs to establish new blood vessels for their maintenance.

Accumulated evidences have shown that both endothelial and non-endothelial cells recruit pericytes in tumor blood vessels through PDGF-B, its receptor (PDGF-Rβ) and VEGF signaling networks in a mouse fibrosarcoma model and in U87MG glioma model [18,19]. Recently, our studies have found that breast tumor cells are capable of modulating the migration of vascular SMCs *in vitro*, and this event is mediated through vascular endothelial growth factor (VEGF)/B-form of platelet-derivative growth factor (PDGF-B) - neuropilin-1 (NRP-1) signaling pathways [20,21]. This study, for the first time to our knowledge, shed light on the molecular interactions of tumor cells with mesenchymal stem cells, and offers new opportunities to improve the understanding of the regulation of pathologic pericytes by cell-cell interactions through successive studies. The main objective of the present work is to extend our initial findings and test the hypothesis that the interaction of tumor cells with mural precursor cells may cause differentiation of precursor cells to PCs (mesenchymal to pericyte transition) and the recruitment/attachment for tumor angiogenesis.

To test this concept, we determined whether different tumor cell-derived conditioned media are able to differentiate and recruit the mesenchymal stem cells to pericytes. We demonstrate that the tumor cell-derived conditioned media are capable of differentiating the stem cells to PCs; ultimately recruiting them to bind with endothelial cells differentially. The studies also reveal that tumor cell-secreted PDGF is the responsible molecule for differentiation as well as for recruitment through a physical interaction with NRP-1.

## Results

### Tumor cell-derived conditioned media (TCM) enhance the proliferation of mesenchymal stem cells

**Figure 1:**
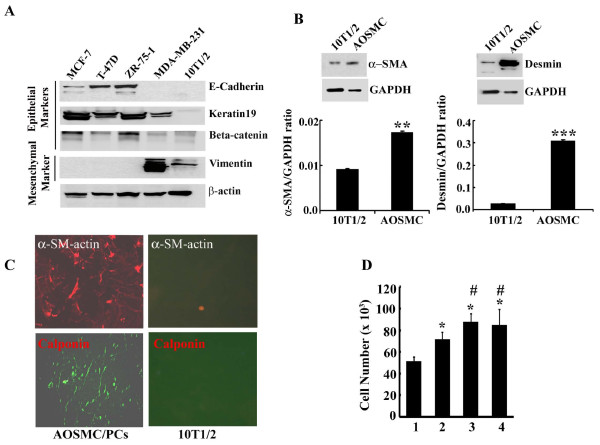
**Molecular characterization and the effect of TCM on proliferation of 10T1/2 mesenchymal stem cells**. **A**. Identification of mesenchymal properties of 10T1/2 cells by determining the expression of epithelial or mesenchymal marker proteins using Western blot analysis. **B**. A comparative study of α-smooth muscle actin (α-SMA) and desmin expression level in 10T1/2 cells and AOSMC by Western blot analysis using specific antibodies. The bar graphs represent the quantitative data (mean ± SEM) from three separate experiments. *P*-values were determined by unpaired student's *t*-test. **p < 0.005 *vs *10T1/2 and ***p < 0.001 *vs *10T1/2. **C**. Detection of α-SMA and calponin in 10T1/2 cells and AOSMC using immunofluorescence. **D**. Effects of different TCM on 10T1/2 cell proliferation. 1, regular media (RM), 2, MCF-7 cells derived conditioned media, 3, MDA-MB-231 cells derived conditioned media and 4, Mia-Paca-2 cells derived conditioned media. *P*-values were determined by unpaired students't-test. *p < 0.01 *vs *RM; #p < 0.05 *vs *MCF-7-CM.

For new blood vessel formation, mesenchymal stem cells need to proliferate. In this experiment, we determined whether tumor cell-derived diffusible factors in the conditioned media are able to increase the mesenchymal stem cell's proliferation rate. To test this, C3H/10T1/2 mouse mesenchymal stem cells were used for this study. These cells were isolated from C3 H mouse embryo pluripotent stem cells and considered as mesenchymal cells because they have the ability to differentiate into a variety of mesodermal cell lineages [22]. Our studies are in agreement with previous findings and indicate that this embryonic cell line is the mesenchymal type. The immuno-Western blot analysis showed that epithelial marker proteins (i.e., E-Cadherin, Keratin 19, Beta-catenin) are absent in these cells while mesenchymal marker, vimentin, is overexpressed (Fig. 1A). Moreover, mural cell markers are either absent or minimally expressed in 10T1/2 cells (Fig. 1B and 1C). These include α-SMA, calponin and desmin. After confirmation of the stem cell behavior, 10T1/2 cells (60% confluent) were incubated with different TCM for 24 hours. Cells were trypsinized and stained with trypan blue for the count in Cellometer Auto T4 (Nexcelom Bioscience, Lawrence, MA 01843). We found that the cell number of the conditioned media incubated 10T1/2 cells increased more significantly than the control, and maximum cell number was increased in the invasive (MDA-MB-231 and PaCa-2) cell line-derived-CM in comparison to the non-invasive (MCF-7) cell line-derived CM (Fig. 1D). In order to confirm whether the induction of cell number was due to the proliferation or inhibition of apoptosis, we determined the apoptosis using cell death ELISA assay. No significant apoptotic cell death was observed in TCM exposed and unexposed cells (data not shown). Collectively, these studies indicate that TCM induced induction of cell numbers is due to the stimulation of cellular proliferation.

### TCM is able to differentiate the mesenchymal stem cells to pericytes

**Figure 2:**
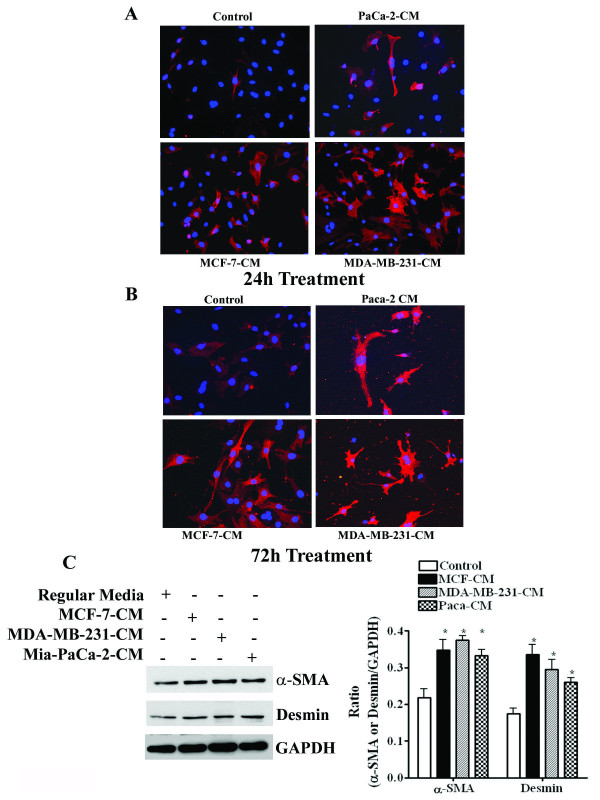
**Mesenchymal to pericyte transition (MPT) by different tumor cell-derived conditioned media (TCM)**. **A-B**. Representative images of MPT of 10T1/2 mesenchymal cells grown in regular media or different tumor cell-derived conditioned media for 24 h or 72 h. Cells are immuno-stained with pericyte-specific marker α-SMA, X200. **C**. Imuno-Western blots of α-SMA and desmin in 10T1/2 cells grown in different TCM for 24 h. The bar graphs represent the quantitative analysis from three different experiments. P-values were determined by unpaired student's-test. *p < 0.01 *vs *control (RM).

The communication between tumor cells and surrounding stroma is a well established phenomenon and this event is very crucial for tumor growth [23-26]. However, tumor cell-induced mesenchymal stem cell (MSC) transition to pericytes and their attachment in contribution to neovascularization has not yet been fully elucidated. Therefore, we investigated whether human tumor cells are able to force the mouse MSC to differentiate to pericytes (PCs). To do so, semi-confluent 10T1/2 MSC were incubated with 10T1/2 media (RM), or different tumor cell (MIA-PaCa-2, MCF-7 and MDA-MB-231)-derived conditioned media (TCM) for 24 and 72 h and fixed and processed for immunofluroscent staining with alpha smooth muscle actin (α-SMA), a positive marker of mural cells [11]. As shown in Fig. 2, α-SMA antibody weakly immuno-reacts with RM-exposed cells as compared to TCM-exposed cells. In TCM-exposed cells, reactions were increased in time dependent fashion. Furthermore, we found that the staining intensity of α-SMA was reliant on the aggressiveness of the tumor cells. For example, MCF-7 non-invasive breast tumor cell-derived CM moderately enhanced α-SMA expression in 10T1/2 cells, while its expression was very strong in invasive cells (MDA-MB-231 and MIA PaCa-2)-derived CM treated cells. The numbers of α-SMA positive cells increased significantly in TCM-exposed cells as compared to RM-exposed cells (data not shown). The morphology of the 10T1/2 cells in the TCM groups was markedly altered to pericytes-like cells that exhibited a slender, bipolar morphology with cellular processes.

To confirm the immunofluorescence staining results, we have determined the status of the αSM-actin and desmin protein level in 10T1/2 cells following incubation with different cell-derived conditioned media for 24 h. We found significant induction of αSM-actin and desmin expression in TCM exposed cells as compared to RM (Fig. 2C).

### PDGF-B induces Mesenchymal-Pericyte-Transition (MPT)

**Figure 3:**
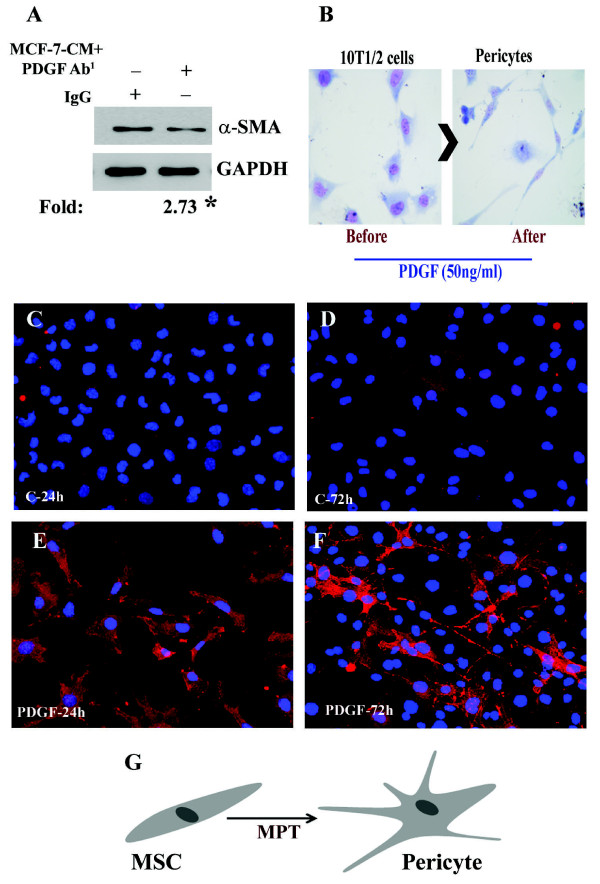
**Tumor cell secreted PDGF-B is involved in MPT induced by tumor cells**. **A**. Representative immuno-Western blot image illustrates the effect of PDGF neutralizing antibody on MCF-7-CM-induced α-SMA expression in 10T1/2 cells. **B**. Representative Giemsa stained Photographs show that mouse mesenchymal stem cells are converted into pericytes by PDGF recombinant protein after 72 h treatment. **C-F**. Representative immunofluorescence images exhibit MPT and α-SMA expression in PDGF-B exposed 10T1/2 cells. **G**. **G**. Schematic illustration of mesenchymal stem cell into pericyte transition.

It is well established that tumor cells secrete different kinds of growth factors and previously we found that Platelet-derived growth factor-AB/BB (PDGF-AB/BB) is one of them [20]. PDGF-AB/BB is required for maintaining the potential biological events. Since we found that human tumor cell-derived conditioned media is able to differentiate the mouse mesenchymal stem cells into pericytes, we investigated whether PDGF-AB/BB existed in the TCM, and is required for the mesenchymal to pericyte transition (MPT). To do so, first, we neutralized the MDA-MB-231-TCM with polyclonal rabbit anti-PDGF-BB antibody (500 ng/ml) or rabbit anti-IgG (negative control) and determined the status of α-SM-actin using Western blot analysis. The expression level of the protein in neutralized samples was significantly reduced by 2.73-fold as compared to negative controls (Fig. 3A). Next, we determined the morphological alterations as well as α-SM-actin expression using an immunoefluorescence assay. To examine this, ~60% confluent 10T1/2 cells were incubated with or without recombinant PDGF-BB (50ng/ml) at three different times and cells were fixed with ethanol. The cells treated for 72 h, were stained with Giemsa solution for morphological studies, which illustrated a differentiation of fibroblast-like structures into thread-like cells (Fig. 3B). The cells treated for 24 and 72 h were also processed for immunofluorescence staining for α-SM-actin to confirm the morphological and molecular changes, respectively (Fig. 3C-3F). The level of α-SM-actin protein was increased in treated cells in a time dependent fashion. The morphological changes can also be seen in fluorescence stained cells. Collectively, the studies indicate that the transition from MSC to PC can be accomplished by PDGF-BB (Fig. 3B-3G).

### NRP-1 is required for PDGF-B-induced MPT

**Figure 4:**
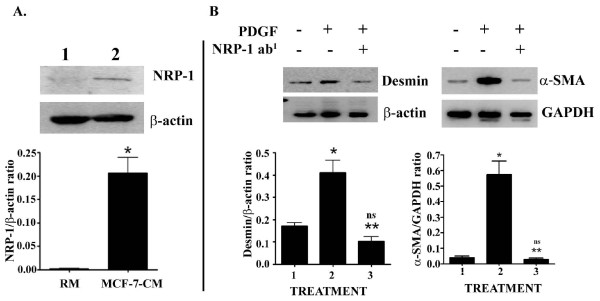
**NRP-1 is required for PDGF-B mediated induction of α-SMA and desmin expression**. **A**. Representative immuno-Western blot shows the effect of MCF-7-CM on NRP-1 expression in 10T1/2 cells. 1, RM and 2, MCF-7-CM. *p < 0.001 *vs *RM. **B**. Detection of the effect of NRP-1 antibody on PDGF-B-induced Desmin and α-SMA expression in 10T1/2 cells. The bar graphs represent quantification of the Desmin and α-SMA of three different experiments, P-values were determined by unpaired students't-test. *p < 0.001 *vs *controls; **p < 0.001 *vs *PDGF treated samples. Ns, non-significant with controls.

Previous studies have manifested a physical interaction of PDGF-BB with NRP-1 for vascular SMC motility for angiogenesis [20]. The objective of this work was to evaluate whether NRP-1 is required for PDGF-BB mediated MPT. To do so, first, we determined the status of NRP-1 in TCM exposed and unexposed 10T1/2 cells. NRP-1 expression was minimally detected in the cell lysate (30 μg) of 10T1/2 cells, but its expression was markedly elevated in MCF-7-CM-exposed 10T1/2 cells (Fig. 4A). Next, we determined the impact of the PDGF-BB recombinant protein on MPT through the detection of tumor blood vessel-pericyte markers, such as Desmin and α-SMA [15,16]. As expected, PDGF significantly increased the expression of both markers in 10T1/2 stem cells (Fig. 4B, lane 2), and these inductions were nullified when cells were pre-exposed to NRP-1 primary antibody (Fig. 4B, lane 3). Collectively, these studies suggest that a physical interaction of PDGF-BB and NRP-1 is crucial in PDGF-BB mediated MPT.

### TCM is able to recruit/attach of 10T1/2 to a capillary-like structure *in vitro *through PDGF-B

**Figure 5:**
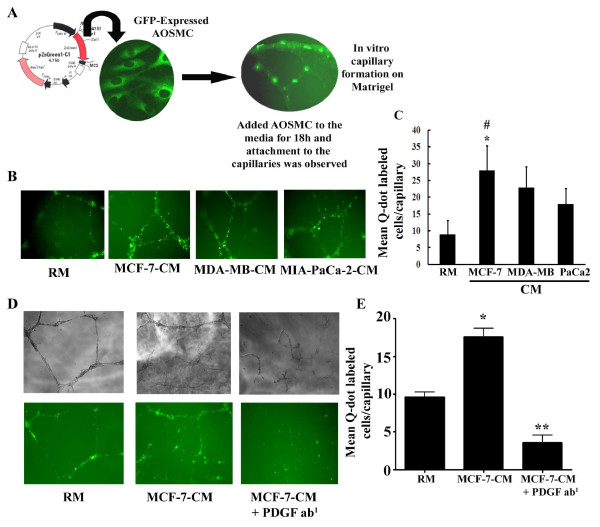
**Recruitment/attachment of vascular SMC and TCM-induced newly formed pericytes from 10T1/2 mesenchymal stem cells on HUVEC-generated capillary-like structures on Matrigel**. **A**. The photograph showing the attachment of GFP (pZsGreen1C1, GFP-producing vector) -transfected AOSMC on the newly formed capillaries by HUVEC on 3 D matrigel. **B**. Differential attachment of Q-dot labeled newly formed pericytes from 10T1/2 cells on the HUVEC generated capillaries by RM or different tumor cells derived conditioned media on 3 D matrigel. **C**. Number of attached Q-dot-labeled newly created pericytes under different tumor cells-conditioned media on capillary-like structures formed by HUVEC on Matrigel. The data are representative mean ± SEM of ten different capillary-like structures. *p < 0.0001 *vs *RM; #p < 0.05 *vs *MDA-MB-CM or PaCa-CM. **D**. Upper panel shows the capillary formation in different treatment conditions, and lower panel shows the attachment of Q-dot labeled 10T1/2 cells on the newly formed capillaries by HUVEC in different treatment conditions on matrigel. RM, Regular Media, MCF-7-CM, MCF-7 cell-derived condition media and MCF-7-CM + PDGF ab^1^, MCF-7-CM neutralized by PDGF antibody. Quantification of attachment of Q-dot-labeled cells using software provided by Nikon fluorescent photo microscope. The represents mean ± SEM of 10 capillaries of each experiment of three different experiments. *P*-values were determined by unpaired students't-test. *p < 0.001 vs RM; **p <0.001 *vs *MCF-7-CM.

In this experiment, we explored whether TCM are able to increase the recruitment/attachment of transitioned pericytes with the capillaries formed by the HUVEC. To do so, a 3 D Matrigel™co-culture was employed. First, HUVEC were allowed to grow on Matrigel to form a capillary-like structure in the presence of GFP-tagged-AOSMC for 18 h and capillary formation was visualized by fluorescent microscopy. Consistent with previous work [27,28], this study showed that like *in vivo*, the AOSMC were able to attach to the capillary-like structures under *in vitro *conditions (Fig. 5A). Next, we evaluated whether TCM-induced mesenchymal-pericyte transition cells are capable of attaching to the capillary-like structures *in vitro*. To test this, HUVEC and Q-dot labeled 10T1/2 mouse mesenchymal stem cells were seeded on the matrigel, and their associations were observed after 20 h when the *in vitro *capillary-like structures are formed. The pattern of the tube-like structure formations in various TCM is different than regular media. The number of capillary-like structures was significantly higher in TCM. Moreover, the recruitment and attachment of the newly formed TCM-induced-pericytes were significantly increased under TCM as compared to RM (Fig. 5B and 5C). The recruitment and attachment of pericytes vary significantly within TCM. The effect was more pronounced in MCF-7 cell-derived conditioned media. Finally, we needed to determine if PDGF-BB was involved in the TCM-mediated recruitment event. To do so, HUVEC and Q-dot labeled 10T1/2 cells were grown on the matrigel containing RM, MCF-7-CM or PDGF-BB antibody-pre-exposed-MCF-7-CM for angiogenesis assay. As expected, MCF-7-CM markedly increased the *in vitro *capillary-like structure and the attachment of Q-dot-labeled cells as compared to RM (Fig. 5B &5C). In contrast, PDGF-B antibody-pre-exposed-MCF-7-CM was unable to either induce capillary-like structure on matrigel or induce attachment of labeled cells to the capillaries (Figs. 5D and 5E).

### TCM is able to recruit/attach of 10T1/2 cell to the newly formed capillaries *in vivo*

**Figure 6:**
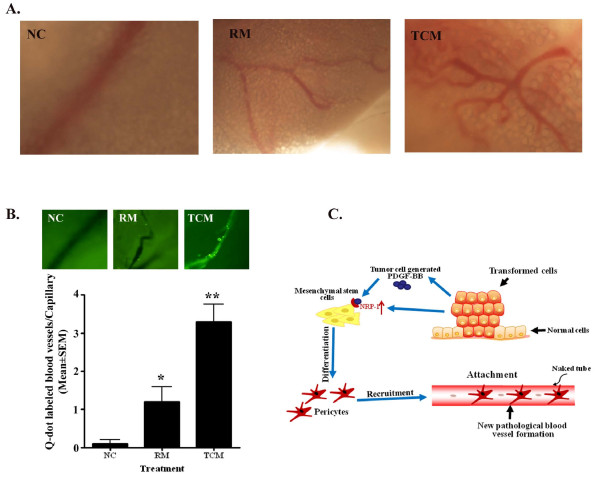
**TCM-induced tumor angiogenesis and recruitment/attachment of Q-dot-labeled newly formed pericytes on blood vessels formed on gel-foam inserted into mice**. **A**. Representative images show the effect of MCF-7-CM on *in vivo *angiogenesis. Gelfoams, soaked with water (NC), regular media (RM) or tumor cell-derived media (TCM), were transplanted into the subcutis of mice and angiogenesis was viewed under the microscope. **B**. The photograph showing the recruitments of q-dot labeled pericytes on in vivo angiogenesis in three different conditions [Gelfoam soaked with water (NC), regular media (RM) and tumor-cell-derived condition media (TCM)]. The bar graph represents the number of Q-dot-labeled blood vessels per field in different treatment conditions. The data represent mean ± SME of three different experiments. *p of <0.0012 vs negative controls (NC); **p <0.0001 *vs *RM. **C**. Diagrammatic illustrations of signaling pathways involved in tumor cells-generated PDGF-B-induced differentiation of pericytes from mesenchymal stem cells. The studies demonstrate that PDGF-B is responsible for the differentiation of pericytes from mesenchymal stem cells through PDGF-B-NRP-1 signaling pathway.

We investigated whether TCM were able to induce tumor angiogenesis under *in vivo *condition. To do so, an *in vivo *foam-gel angiogenesis assay was performed. This assay showed that capillary formation was markedly enhanced on the MCF-7-CM-soaked gel foam as compared to water or RM soaked gel-foam (Fig. 6A). Next, we determined whether TCM promotes recruitment and attachment of 10T1/2 cells in TCM-induced newly formed blood vessels. Like *in vitro*, TCM significantly increased the recruitment and attachment of labeled 10T1/2 cells on newly formed blood vessels on the gel-foam (Fig. 6B).

## Discussion

Differentiation of mural precursor cells to vascular SMCs/PCs and their recruitment are the fundamental events for the maturation of both normal and tumor blood vessels created from nascent vessels [3,20,29]. One aspect that is clear from our previous studies is that tumor cells can cross-talk with vascular SMCs. The present study further described an important issue of whether tumor cells have any roles in PC biology, most specifically, enhancement of the differentiation of mesenchymal stem cells to pericytes and subsequent recruitment/attachment of PC for tumor angiogenesis through a specific molecular networking circuit.

The initial objective of the present work was to establish the working hypothesis that the interaction between tumor cells and mural precursor cells causes the differentiation of precursor cells to PCs, which ultimately proliferate and recruit to establish a mature and durable vessel. We found that when 10T1/2 cells, C3 H mouse embryonic mesenchymal stem cells (C3H/10T1/2) as characterized by us and others [22] (Fig. 1), were grown in different breast and pancreatic cancer cell-conditioned media for 2-4 days, the stem cells differentiate into pericytes with abnormally high expression of α-SMA along with Desmin (Fig. 2). These features are identical to the pericytes on tumor vessels [15,16]. Thus, we can anticipate that tumor cells have the capability to generate specific signals for mesenchymal to pericyte transitions.

Our next goal was to identified the signaling networks involved in tumor cell-induced MPT. The pericytes can originate from various cell lineages [14,30] and commonly come from mesenchymal stem cells [14,31]. There are two important molecular signaling pathways that are involved in the development of pericytes [32], TGF-β and PDGF-B signaling pathways [14,33,34]. Previous studies have shown that embryonic stem cells are able to differentiate into endothelial cells if they are exposed to VEGF, while they can be differentiated into pericytes in presence of PDGF-B [35-37]. In our *in vitro *model, we found that tumor cell-secreted PDGF-B plays a critical role in MPT (Figs.3 and 4). Most importantly, we found that the tumor cell-secreted PDGF-B-induced MPT event is mediated through NRP-1 (neuroplin-1), which is a co-receptor for semaphorins with key roles in axon guidance, a docking receptor for VEGF_165 _[38,39] and exhibits a physical interaction with PDGF-B in vascular SMC to enhance their migration [20,38].

One of the key steps of the termination of angiogenesis is the incorporation of pericytes/vascular SMCs into the newly formed vessels [13,40,41]. We explored if tumor cells can enhance the recruitment and incorporation of pericytes into the newly formed endothelial tubes under *in vitro *and *in vivo *setups. The studies showed that in addition to induction of MPT, the tumor cell-generated signals including PDGF-B are able to increase the recruitment and attachment of newly differentiated pericytes into the blood vessels (Figs. 5 and 6). However, the impact of different tumor cell lines that cause pericytes to attach to blood vessels is different. Less aggressive cell lines, such as MCF-7 non-invasive breast tumor cell-generated CM caused more abundant recruitment/attachment of pericytes to the blood vessels as compared to aggressive cell lines (i.e., MDA-MB-231 and MIA-PaCa-2)-generated CM (Fig. 5). Recent studies reveal that pericytes limit metastatic spread in pancreatic β-islet cell tumorigenesis in PDGFRb^ret/ret ^mice [42] and in patients with colorectal cancer [43]. In agreement with these findings, less abundant recruitment of pericytes by aggressive tumor cell-CM may correlate with metastatic spread of these cells. Because MCF-7, MDA-MB-231 and MIA-PaCa-2 cells highly expressed PDGF-B but the CM of later two tumor cell lines were unable to facilitate pericytes to attach more abundantly to the capillary-like structures as compared to MCF-7-CM, these studies indicate that some additional factor(s) is essential for proper adhesion of pericytes to the vessels, which is either missing or inhibited by aggressive cancer cells for metastatic spread. Further studies are warranted. Finally, the studies also show that PDGF-B signaling not only influences MPT and recruitment of pericytes but also affects *in vitro *capillary stability by unknown mechanisms (Fig. 5D, last panel).

## Conclusions

In conclusion, this work, as depicted in Fig. 6C, provides direct evidence that tumor cells enhance the mesenchymal to pericyte transition event for the recruitment and adhesion of pericytes on newly formed blood vessels to terminate the tumor angiogenesis process. These multistep events are apparently mediated by tumor cell-secreted PDGF-B signaling molecule. NRP-1 may play a critical role in this event. We anticipate a direct link between the disparity in recruitment of pericytes by various tumor cells and their metastatic potency. In support of this study, targeting tumor cell-secreted PDGF-B or NRP-1 in pericytes by inhibitors may efficiently diminish the tumor angiogenesis, tumor growth and metastatic spread.

## Materials and methods

### Cell culture

The mouse embryonic mesenchymal stem cells, C3H/10T1/2, non-invasive breast tumor cells MCF-7, T-47 D and ZR-75-1, invasive breast cancer cells MDA-MB-231and invasive pancreatic carcinoma cells MIA-PaCa-2, were obtained from American Type Culture Collection (ATCC, Manassas, VA) and grown in Dulbecco's modified Eagle's medium (DMEM, Sigma Chemical Co, St. Louis, MO) supplemented with 10% fetal calf serum and antibiotics, in a humidified incubator at 37°C in an atmosphere containing 5% CO_2 _and 95% O_2. _Human aortic smooth muscle cells (AOSMC) and HUVEC (human umbilical vein endothelial cells) were purchased from Cambrex and grown in smooth muscle cells basal media (SmBM) with various growth factors (SmGM-2, i.e., insulin, FGF, EGF and 2% serum) and EGM-2 bullet kit (EBM-2, the basal medium supplemented with growth factors and 5% serum) respectively.

### Animals

FVB/N mice (6-8 weeks of age), purchased from Taconic (Hudson, NY) were housed in autoclaved cages fitted with high efficiency filter-tops and with autoclaved bedding. The animals were fed irradiated Purina chow. The room was kept at 25°C with a 12-h light-dark cycle. The animal studies were carried out as per the guidelines established in the *Guide for the Care and Use of Laboratory Animals*, US Department of Health and Human Resources (NIH 1985) and VA Animal Care facilities.

### Reagents

B-forms of PDGF protein and Giemsa were purchased from Sigma Chemical Co. Polyclonal rabbit anti-PDGF-BB, mouse monoclonal alpha smooth muscle-actin and Desmin antibodies were purchased from abCam (Cambridge, MA). Goat-anti mouse E-cadherin and β-Catenin and Goat-anti mouse Cytokeratin-19 and Vimentin were obtained from BD Transduction and Labvision (Neomarker) respectively. A Qtracker cell labeling kit was purchased from Invitrogen (Molecular Probes, Eugene, Oregon). Matrigel was purchased from BD Biosciences.

### Preparation of tumor cell-Conditioned Media (TCM)

The procedure of preparation was the same as previously described [20]. Briefly, MCF-7, MDA-MB-231 and MIA-PaCa-2 cells were grown in DMEM media for 24 hours and collected. They were centrifuged for 10 min at 2000 rpm at 4°C to remove the cells or cell debris. After, centrifugation, media were collected and used for the experiments.

### Cell Count

~70% confluent 10T1/2 cells were incubated with different tumor cell-derived condition media for 24 hours. Cells were trypsinized after 24 hours and stained with trypan blue for the count in Cellometer Auto T4 (Nexcelom Bioscience, Lawrence, MA 01843).

### Immunofluorescence

Briefly, ~60% 10T1/2 confluent cells that were grown in a one-well slide chamber were incubated with or without PDGF-BB (Platelet-derived growth factor-BB) or different tumor conditioned media (MCF-7, MDA-MB-231 and MIA-PaCa-2) for 24 and 72 hours at 37°C and fixed with methanol. The slides were then permeabilized with Triton-X-100 and incubated with blocking solution (Histostain kit, Zymed Laboratories, CA) for 10 mins at room temperature, and cells were reacted with mouse monoclonal alpha smooth muscle actin antibody overnight at 4°C. After being washed with PBS, the cells were incubated with goat anti-mouse IgG fluorescent conjugated secondary antibody for an hour at room temperature (1:1000 dilutions, Alexa Fluor 594, Molecular Probes). Finally, the washed cells were mounted in PBS-glycerin and examined under a fluorescent microscope.

### Western blot analysis

The Western blot analysis was performed in cell lysates treated with different cells derived conditioned medium according to the method described previously [20]. Cell lysates were obtained by adding lysis buffer containing 50 mM Tris-Cl, pH-8.0, 0.1% SDS, 150 mM NaCl, 1% Nonidet P-40 and protease inhibitor cocktail including 1 μg/ml of Aprotinin, 1 μg/ml leupepsin and 1 mM PMSF and centrifuged at 4°C. The supernatants were collected and protein concentrations were measured with coomassie blue reagent assay (Bio-Rad, Richmond, CA). Equal amounts of protein (10 μg) were subjected to 7.5 - 10% SDS-PAGE and blotted onto a nitrocellulose membrane. Membranes were incubated with specific antibodies anti-mouse monoclonal smooth muscle actin overnight and HRP-conjugated secondary antibodies were incubated for 30 min at room temperature. Signals were detected with Super Signal Ultra Chemiluminescent substrate (Pierce, Rockford, IL) by using ID Image Analysis software Version 3.6 (Eastman Kodak Company, Rochester, NY).

### Neutralization assay

To determine the identity of the growth factor in the tumor cell-derived condition media that were mediating the conversion of stem cells into PCs, the media, conditioned by semi-confluent MCF-7, MDA-MB-231 and MIA-PaCa-2 cells, were preincubated overnight at 4°C with different concentrations (i.e., 200 and 500 ng/ml) of a neutralizing polyclonal antibody against PDGF-BB. The semi confluent mesenchymal stem cells, 10T1/2, were then incubated with regular medium (as a control), tumor cell-derived conditioned media, neutralizing media [tumor cell-derived condition media (CM) neutralized with anti-PDGF-BB antibody and rabbit polyclonal IgG (as a negative control)] for 24 hours at 37°C. After that, cell lysates were collected to perform the Western blot analysis for the detection of the expression level of anti-smooth muscle actin.

### *In vitro *angiogenesis and Binding assay

Approximately 80% confluent 10T1/2 cells were labeled with Qtracker cell labeling kit (highly fluorescent Q-dot nanocrystals) obtained from Invitrogen (Molecular Probes). For 3 D cultures, which has been described earlier [44], Matrigel (150 μl) was polymerized in an 8-well chambered slide. After polymerization, endothelial cell-specific media or tumor cell-generated conditioned media were added. HUVEC and labeled 10T1/2 cells (10,000 cells/well) were seeded into each well and incubated for approximately 20 h which determined the binding efficiency of 10T1/2 cells with capillary-like structure generated by HUVEC in regular media or different tumor cell-derived condition media. Quantification of the number of capillary-like structures and attached Q-dots were carried out using the NIS Elements software program attached with the Nikon photographic fluorescence microscope.

### *In vivo *Angiogenesis Assay

The Gelfoam-implantation angiogenesis assay was performed according to the previous method [45,46]. Briefly, three sets of FVB/N mice (6-8 weeks old; four in each set) were anesthetized immediately before implantation of Gelfoam (Gelfoam^®^, Pharmacia & Upjohn Company, NY, USA). Gelfoam (8 × 8 mm), presoaked with autoclaved water, regular media or TCM, was transplanted subcutaneously in mice. The transplanted mice were maintained for 5-6 days. In the mean time, Q-dot labeled 10T1/2 cells were cultured in regular media or TCM. After a maintenance period, labeled 10T1/2 cells (2 × 10^5^) were injected subcutaneously near implanted Gelfoam according to the experimental set-up. The implanted Gelfoam was taken out carefully after 2 days, and the angiogenesis was detected and quantified using an inverted fluorescence microscope. The protocol has been depicted in Additional file 1: Fig. S1.

### Statistical Analysis

All experiments were performed in triplicate for each of the observations. Each of the data represents the mean ± SE from the three separate experiments. Statistical analysis was performed between the two groups of data by an unpaired Student's *t*-test. A *P*-value less than 0.05 were considered as statistically significant.

## Competing interests

The authors declare that they have no competing interests.

## Authors' contributions

KD, SKB and SB initiated the project and designed the study. KD and GD performed all the treatments and the immunofluorescence. KD, MM, IH and SM mainly were involved in western blot analysis. KD has assisted with the in vitro angiogenesis assay. IH with the help of SM performed the in vivo angiogenesis assay. All authors helped in discussing, reading and proofreading the final manuscript.

## Supplementary Material

Additional file 1 **Figure S1 - Diagrammatic illustration of Gelfoam-implantation angiogenesis assay**. FVB/N mice (6-8 weeks old; four in each set) were anesthetized with Ketamine HCL (100 mg/ml/kg) and Xylazine (20 mg/ml/kg) immediately before implantation of Gelfoam (Gelfoam^®^, Pharmacia & Upjohn Company, NY, USA). Gelfoam was cut into small Pieces (8 × 8 mm), presoaked with autoclaved water, regular media or Tumor cell-conditioned media (TCM). Presoaked Gelfoam was transplanted subcutaneously on left or right flank of mice. The transplanted mice were maintained for 5-6 days. In the mean time, Q-dot labeled 10T1/2 cells were cultured in regular media or TCM. After maintenance period, labeled 10T1/2 cells (2 × 10^5^) were injected subcutaneously near implanted Gelfoam according to the experimental set-up. The implanted Gelfoam was taken out carefully after 2 days and the angiogenesis was detected and quantified using inverted fluorescence microscope. Click here for file
