# Supplementary figures and images for: Tumor cell-derived PDGF-B potentiates mouse mesenchymal stem cells-pericytes transition and recruitment through an interaction with NRP-1
Source: Mol Cancer. 2010 Aug 5;9:209. doi: 10.1186/1476-4598-9-209 (PMC2922194; doi:10.1186/1476-4598-9-209)

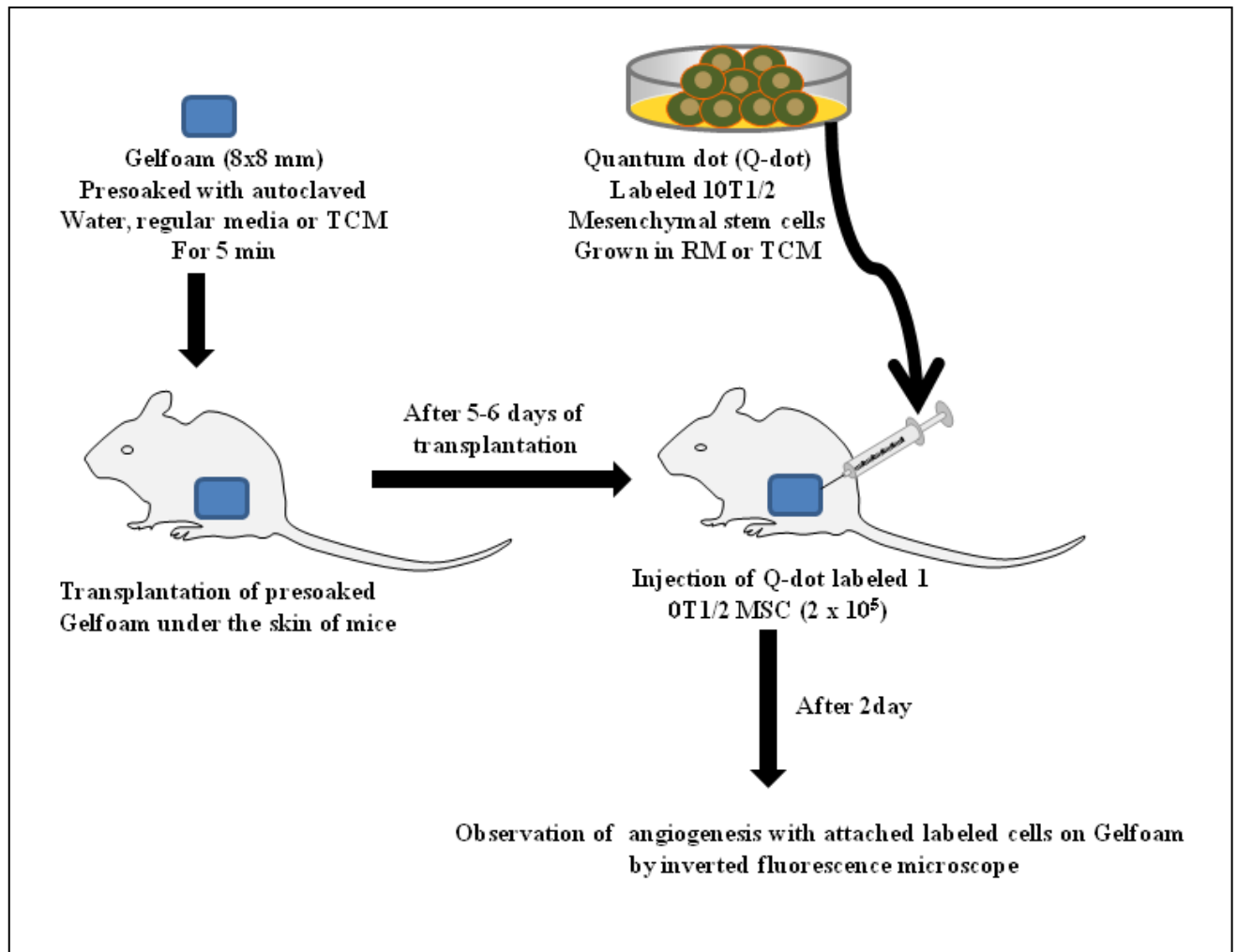

Supplement: Additional file 1 — Figure S1 - Diagrammatic illustration of Gelfoam-implantation angiogenesis assay. FVB/N mice (6-8 weeks old; four in each set) were anesthetized with Ketamine HCL (100 mg/ml/kg) and Xylazine (20 mg/ml/kg) immediately before implantation of Gelfoam (Gelfoam®, Pharmacia & Upjohn Company, NY, USA). Gelfoam was cut into small Pieces (8 × 8 mm), presoaked with autoclaved water, regular media or Tumor cell-conditioned media (TCM). Presoaked Gelfoam was transplanted subcutaneously on left or right flank of mice. The transplanted mice were maintained for 5-6 days. In the mean time, Q-dot labeled 10T1/2 cells were cultured in regular media or TCM. After maintenance period, labeled 10T1/2 cells (2 × 10^5^) were injected subcutaneously near implanted Gelfoam according to the experimental set-up. The implanted Gelfoam was taken out carefully after 2 days and the angiogenesis was detected and quantified using inverted fluorescence microscope. [file 1476-4598-9-209-S1.PDF]
